# Decreased alpha-band oscillatory brain activity prior to movement initiated by perception of fatigue sensation

**DOI:** 10.1038/s41598-019-40605-7

**Published:** 2019-03-08

**Authors:** Akira Ishii, Takashi Matsuo, Chika Nakamura, Masato Uji, Takahiro Yoshikawa, Yasuyoshi Watanabe

**Affiliations:** 1 Department of Sports Medicine, Osaka City University Graduate School of Medicine, 1-4-3 Asahimachi, Abeno-ku, Osaka, 545-8585 Japan; 2 RIKEN, Center for Biosystems Dynamics Research, 6-7-3 Minatojima-minamimachi, Chuo-ku, Kobe, Hyogo, 650-0047 Japan

## Abstract

Fatigue is a health problem prevalent in modern societies. Fatigue sensation plays an important role as a biological alarm urging rest to maintain homeostasis, and clarifying the neural mechanisms related to fatigue sensations by which we decide to engage in rest is therefore essential. This study enrolled healthy male volunteers and showed that the decrease in alpha-band power as assessed by magnetoencephalography of the left Brodmann’s area (BA) 6 before perception of fatigue when a button-press based on the level of fatigue was required was smaller than that before perception of the intention to move when a voluntary button-press was required. In addition, the decrease of alpha-band power in the left BA 6 before the perception of fatigue was not altered compared with that in the right BA 6 when a button-press based on the level of fatigue was required. These results suggest that the button-press based on the perception of fatigue is not prepared before the perception of fatigue. These findings will advance the understanding of the neural mechanisms related to subjective feelings such as fatigue sensation.

## Introduction

Fatigue is a health problem prevalent in modern societies^1–5^ and the accumulation of fatigue caused by overwork can even cause death^6,7^. Ensuring adequate rest and preventing the accumulation of fatigue are thus important.

Fatigue is defined as a decline in the ability to perform or in the efficiency of performing mental and/or physical activities caused by excessive mental or physical activity or disease. Fatigue is frequently accompanied by a peculiar sense of discomfort, a desire to rest, and a decline in motivation, referred to as fatigue sensation^8^. Fatigue sensation is thought to play an important role as a biological alarm urging the individual to rest to maintain homeostasis^9^. Clarifying the neural mechanisms related to fatigue sensation by which we engage in rest to avoid disrupting homeostasis is therefore key to devising methods for preventing the accumulation of fatigue.

The neural mechanisms promoting rest based on the subjective perception of fatigue are doubtlessly important for avoiding the accumulation of fatigue. However, whether the belief of individuals that they decided to engage in rest based on their subjective feeling of fatigue sensation is true actually remains unclear (i.e., whether awareness of the fatigue sensation is essential to the decision, or whether the perception of fatigue sensation is the direct cause of the decision). In other words, the possibility must be considered that the decision an individual believes they have made based on the perception of fatigue has already been made before conscious perception of the fatigue. If the decision that the individual believes they have made based on the perception of fatigue has indeed already been made before perception of fatigue, then the belief that the decision was made based on perception of fatigue is illusory. Therefore, it is of great interest to examine whether the neural activity preceding perception of fatigue is observed when a button-press based on the perception of fatigue is required.

Some kind of brain activity has been shown to take place before the onset of self-initiated activity: a slow negative shift in electrocortical activity as assessed by electroencephalography (EEG), termed Bereitschaftspotential or readiness potential (RP), is observed prior to voluntary movement^10^. This readiness potential starts 1.5 s or more before the onset of voluntary movement and enhances rapidly around 400 ms before onset of the movement. The existence of the RP has been interpreted as neural activity related to preparation for a voluntary movement that is necessary to be ready for the movement (i.e., neural activity preparatory to activity)^10^, neural activity in charge of decision-related or anticipatory processes^11^, or neural activity that affects and/or represents a propensity to act^12^. It is of great interest that the RP is observed far before the moment that the intention to move is perceived when the movement was initiated at a self-chosen moment: the intention to move is usually perceived around 200 to 300 ms before movement onset^13–15^. This observation suggests that the neural activity related to these putative functions such as preparation for a voluntary movement, decision-related or anticipatory processes, or the propensity to act, precede perception of the intention to move when the movement was initiated at a self-chosen moment.

Our interest is in whether perception of fatigue is the direct cause of the decision based on fatigue. The neural activity preceding the onset of a voluntary movement is reportedly assessable by magnetoencephalography (MEG). In an MEG study, the ECD in the contralateral supplementary motor area (SMA) rather than the ipsilateral SMA was observed 1,000 ms before the onset of voluntary finger tapping^16^. In another MEG study, a decrease in alpha-band power was observed before the onset of voluntary finger movement (−500 to 0 ms from movement onset) in the contralateral sensorimotor area and SMA^17^. Taking these findings into consideration, we hypothesized that the decrease in alpha-band power in the contralateral SMA would be observed prior to perception of fatigue if the decision to press the button has already been made before the perception of fatigue when a button-press based on fatigue is required. We therefore performed an experiment in which participants were asked to press a left or right button based on the perceived fatigue with the right index or middle finger, respectively, and assessed the alteration of alpha-band power observed in the contralateral SMA before perception of fatigue using MEG (Task C). We added an experiment in which participants were asked to press a left or right button with the right index or middle finger when they wanted to press to confirm that the decrease in alpha-band power in the contralateral SMA prior to perception of the intention to move was able to be detected in our experiment (Task A), as in previous studies^16,17^. Furthermore, we added another experiment in which participants were instructed to do arithmetic and press a button based on the result of the arithmetic to confirm that the decrease in alpha-band power in the left SMA relative to those in the right SMA was adequately assessed (Task B). The left SMA is reportedly specifically involved in arithmetical tasks and the decrease in alpha-band power in the left SMA is thus expected to be observed prior to subjective recognition of the answer^18,19^. Since the brain regions other than the SMA such as the pre-SMA and premotor area have been reported to play a role in the generation of the neural activity prior to voluntary movement^20^, we assessed the alpha band power in the Brodmann’s area (BA) 6 which includes the SMA, pre-SMA, and premotor area.

## Materials and Methods

### Participants

Nineteen healthy male volunteers with a mean (±standard deviation [SD]) age of 22.7 ± 0.6 years took part in this study. All participants were right-handed according to the Edinburgh Handedness Inventory^21^, and none had any history of mental illness, brain injury, or upper extremity disorder. Current smokers and individuals taking chronic medications that could affect the central nervous system were excluded. The Ethics Committee of Osaka City University approved the study protocol (approval number 3770). All participants provided written informed consent to participate in this study in accordance with the principles of the Declaration of Helsinki and the Japanese Ethical Guidelines for Medical and Health Research Involving Human Subjects.

### Experimental tasks

Three types of tasks (Tasks A, B, and C) were performed in randomized order on three different days. All tasks were designed to identify the onset of awareness of the intention to press the left or right button and the time at which either button was pressed. Participants were asked to perform task trials projected on a screen by a projector (PG-B10S; SHARP, Osaka, Japan) using an MEG-compatible response device (HHSC-164-L; Current Designs, Philadelphia, PA). To assess alterations in oscillatory neural activity prior to the button-press, MEG recordings were performed over the task trials.

**Figure 1:**
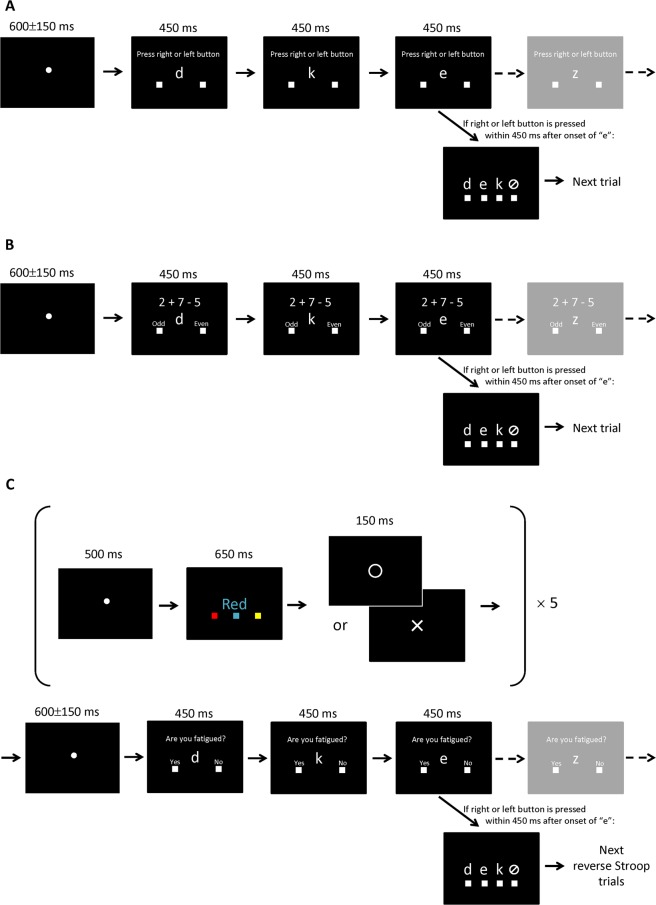
Experimental tasks. Three types of tasks were performed in randomized order on three different days. (**A**) In Task A, the letter presented on the center of the screen was updated every 450 ms and the participant was instructed to press the right or left button using the right index or middle finger, respectively, at the time they wanted to press either button as quickly as possible. They were also asked to memorize the letter presented at the time they wanted to press either button and to report the letter by selecting one of three letters presented on the screen. (**B**) In Task B, simple arithmetic tasks were presented on the screen in addition to the letter, which was updated every 450 ms. The participant was instructed to perform the arithmetic and to answer whether the result of the arithmetic was odd or even by pressing the left or right button, respectively, as quickly as possible when the answer was calculated. They were also asked to memorize the letter presented at the time the answer was calculated and to report the letter by selecting one of three letters presented on the screen. (**C**) In Task C, the participant was asked to perform a reverse Stroop task, pressing the button corresponding to the color denotated by the word presented on the screen as quickly and accurately as possible. After every five reverse Stroop task trials, they were asked to answer whether they were fatigued or not at that time by pressing the left or right button, respectively, as quickly as possible when they perceived fatigue. The letter presented on the center of the screen was updated every 450 ms. They were also asked to memorize the letter presented at the time they perceived fatigue and to report the letter by selecting one of three letters presented on the screen. In Tasks A, B, and C, the participant was asked to press the rightmost button if they were unable to memorize the letter, which was updated every 450 ms.

In Task A, the letter presented in the center of the screen was updated every 450 ms and the participant was instructed to press the right or left button as quickly as possible using the right index or middle finger, respectively, at the time they wanted to press either button (voluntary key press). They were also asked to memorize the letter presented at the time they wanted to press the button and to report the letter by selecting one of the three letters presented on the screen just after voluntary key press (Fig. 1A). A sequence of 80 presentations constituted one session, and five sessions were performed in a row with about 5 min between sessions.

In Task B, simple arithmetic tasks were presented on the screen in addition to the letter which was updated every 450 ms. Participants were instructed to perform the arithmetic and to answer whether the result of the arithmetic was odd or even by pressing the left or right button, respectively, as quickly as possible when they calculated the answer. They were also asked to memorize the letter presented at the time they calculated the answer and to report that letter by selecting one of three letters presented on the screen, just as in Task A (Fig. 1B). A sequence of 80 presentations constituted one session, and five sessions were performed in a row with about 5 min between sessions.

In Task C, participants were asked to perform the reverse Stroop task, which was identical to that used in our previous study^9^. The participant was required to press the button corresponding to the name of the color denotated by the word presented on the screen, as quickly and accurately as possible. After every five reverse Stroop task trials, the participant was asked to answer whether they were fatigued or not at the time by pressing the left or right button, respectively, as quickly as possible after perceiving fatigue. The letter presented on the center of the screen was updated every 450 ms, as in Task A. They were also asked to memorize the letter that was presented at the time they perceived the fatigue and to report that letter by selecting one of three letters presented on the screen, just as in Task A (Fig. 1C). A sequence of 80 presentations constituted one session, and five sessions were performed in a row with about 5 min between sessions.

In Tasks A, B, and C, the participant was asked to press the rightmost button if they were unable to memorize the letter, which was updated every 450 ms.

### MEG recording and magnetic resonance (MR) imaging overlay

MEG was recorded using a 160-channel whole-head-type MEG system (MEG vision; Yokogawa Electric Corporation, Tokyo, Japan). The sensor and reference coils were gradiometers with a 15.5-mm diameter and 50-mm baseline, and the two coils were separated by 23 mm. The sampling rate was 1,000 Hz and data were high-pass filtered at 0.3 Hz.

Anatomical MR imaging was performed to permit registration of magnetic source locations with the respective anatomical locations (Philips Achieva 3.0 TX; Royal Philips Electronics, Eindhoven, the Netherlands). Five adhesive markers (Medtronic Surgical Navigation Technologies, Broomfield, CO) were attached to the skin of the head before the MR scanning and MEG data were superimposed on MR images using information obtained from these markers and MEG localization coils.

### MEG analyses

The magnetic noise that originated from outside the magnetically shielded room was eliminated by subtracting the data obtained from reference coils using specialized software (MEG 160; Yokogawa Electric Corporation). Epochs of raw MEG data that included artifacts were visually identified and excluded before analysis. To identify the alterations in oscillatory neural activity preceding the button-press, spatial filtering analysis of MEG data was performed^22–24^. MEG data were bandpass filtered at 8–13 Hz by a finite impulse response filtering method using Brain Rhythmic Analysis for MEG software (BRAM; Yokogawa Electric Corporation), and then the location and intensity of cortical activities were estimated using BRAM, which uses a narrow-band adaptive spatial filtering algorithm^25,26^. Voxel size was set at 5.0 × 5.0 × 5.0 mm. For each task, only those trials in which the reported onset of the intention to press the button (i.e., perception of the intention to press the button in Task A, recognition of parity of the answer in Task B, and perception of fatigue in Task C) was within the temporal window of −300 to 0 ms from the onset of the button-press were included in the analysis. Those trials in which the button was pressed within 150 ms from the onset of presentation of the letter reported by the participant were excluded from analyses, because the intention to move is reportedly perceived around 200 to 300 ms before movement onset^13–15^; a latency between perception and button-press less than 150 ms may thus be attributed to a failure to report the letter or press the button. In addition, trials in which the time between the end of fixation and button-press (i.e., pre-trigger period) was less than 1,000 ms were excluded from analysis. The oscillatory powers of MEG data from −900 to −750 ms, −750 to −600 ms, −600 to −450 ms, and −450 to −300 ms before the button-press were calculated relative to that in the baseline period, which corresponded to the last 300 ms of presentation of the fixation. In Task C, both the trials with answer “yes” and those with answer “no” were included in the analysis. The obtained oscillatory power of MEG data was transformed into the Montreal Neurological Institute T1-weighted image template^27^ using SPM software (SPM8; Wellcome Department of Cognitive Neurology, London, UK) implemented in Matlab (Mathworks, Natick, MA) and the anatomically normalized MEG data were filtered with a Gaussian kernel of 20 mm (full-width at half-maximum) in the x-, y-, and z-axes. The alterations in oscillatory power at voxels in the right and left BA 6 were extracted using Marsbar ROI tool box for SPM (^28^; https://sourceforge.net/projects/marsbar/) and mean values of voxels in the left and right BA 6 were calculated.

### Statistical analyses

Values are presented as mean and SD unless otherwise stated. Two-way repeated-measures analysis of variance (ANOVA) was performed to assess the effects of task and time, and the effects of laterality (i.e., right or left) and time on alterations in oscillatory brain activity. A paired *t*-test with Bonferroni correction was used to compare alterations in oscillatory brain activity in the left BA 6 between Tasks A and B and between Tasks A and C, and to compare alterations to oscillatory brain activity in the left BA 6 with those in the right BA 6 in Tasks A, B, and C. All *P*-values were two-tailed and values less than 0.05 were considered statistically significant. All statistical analyses were performed using the IBM SPSS version 21.0 software package (IBM, Armonk, NY).

## Results

### Number of trials left for analyses

Nineteen healthy male volunteers were participated in this study. Two participants were excluded from analysis because they fell asleep during the task trials. It has been reported that the precision for the peak intensity estimated by spatial filtering algorism of MEG data depends on the number of time points included in the data analyzed^29^: The precision increases as the number of time points increases and reaches almost plateau at 6,000 time points. The sampling rate of MEG recording in our present study was 1,000 Hz and thus, to ensure that MEG data in each task included more than 6,000 time points (i.e., 150 ms × 40 trials), participants who showed MEG data with less than 40 trials in either task were excluded from analyses^29^. As a result, MEG data from nine participants were analyzed. The numbers of trials in which the time between reported onset of the intention to press the button and the button-press (i.e., delay) was not concluded to be within 300 ms (indicated as “Delay > 300 ms” in Table 1), in which the button was pressed within 150 ms from the onset of presentation of the letter reported by the participant, in which the time between end of fixation and button-press (i.e., pre-trigger) was less than 1,000 ms, or in which the final number of trials left after exclusion of trials contaminated with electromagnetic noise are shown in Table 1. These numbers did not differ significantly between Tasks A and B or between Tasks A and C (paired t test without multiple comparison).

**Table 1:** Numbers of trials excluded from analysis and retained for final analysis.

No.	Task A				Task B				Task C			
	Delay > 300 ms	Delay < 150 ms	Pre-trigger < 1000 ms	Final number	Delay > 300 ms	Delay < 150 ms	Pre-trigger < 1000 ms	Final number	Delay > 300 ms	Delay < 150 ms	Pre-trigger < 1000 ms	Final number
2	85	186	0	73	124	140	7	117	114	165	6	57
3	141	138	8	49	143	123	24	71	129	125	13	59
4	153	114	60	49	155	119	3	91	140	125	16	63
9	135	137	0	98	141	124	0	87	129	137	0	98
10	139	127	5	99	157	118	8	101	135	143	10	86
12	141	137	0	82	146	131	3	101	141	111	7	81
13	131	126	1	99	165	120	0	61	185	84	0	79
18	145	129	27	41	164	104	29	66	142	118	9	60
19	195	97	4	64	152	114	10	87	164	101	61	44

Delay: Maximum time between perception of the intention to press the button and button-press.

Pre-trigger: Time between end of fixation and button-press (pre-trigger period).

### Decrease in alpha-band power in the left BA 6 prior to button-press

**Figure 2:**
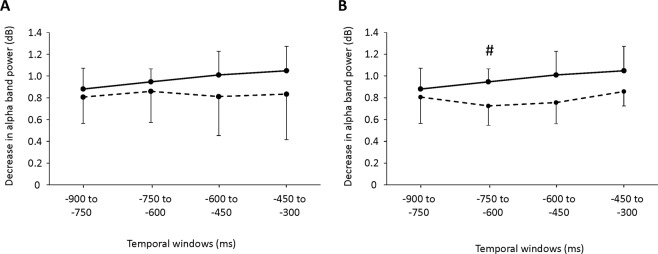
The decrease in alpha-band power observed in the left Brodmann’s area (BA) 6 in Tasks (**A**–**C**). (**A**) Decreases in alpha-band power observed in the left BA 6 in Task A (solid line) and in Task B (dotted line). (**B**) The decrease in alpha-band power observed in the left BA 6 in Task A (solid line) and Task C (dotted line). The alpha-band power from −900 to −750 ms, −750 to −600 ms, −600 to −450 ms, and −450 to −300 ms before the button-press were calculated relative to that in the baseline period, which corresponded to the last 300 ms of presentation of the fixation. ^*#*^*P* < 0.10, paired *t*-test with Bonferroni correction between Task A and Task C at each temporal window.

Two-way repeated-measures ANOVA was performed to assess changes in the decrease of alpha-band power prior to button-press in the left BA 6 between Tasks A and B and between Tasks A and C (Fig. 2). Neither significant main effects nor significant interactions were identified between Tasks A and B (Fig. 2A). A main effect of task was identified between Tasks A and C [F(1, 8) = 6.153, *P* = 0.038] (Fig. 2B). No main effect of time or interaction between Tasks A and C was apparent. The sample size necessary to detect the difference of the decrease of alpha-band power in the left BA 6 between Task A and Task B was calculated by using a software (G*Power)^30,31^. The alpha and beta error were set at 0.05 and 0.1, respectively, and the effect size was calculated based on the results of the two-way repeated-measure ANOVA performed for the difference of alpha-band power in the left BA 6 between Task A and Task C. The total sample size necessary to detect the difference of the decrease of alpha-band power in the left BA 6 between Task A and Task B was 6.

The decrease in alpha-band power in the left BA 6 in Task A tended to be greater than in Task C in the temporal windows of −750 to −600 ms (*t*_8_ = 1.886; *P* = 0.096, paired t test with Bonferroni correction; Fig. 2B).

### Comparison of the decrease in alpha-band power between left and right BA 6 prior to button-press

**Figure 3:**
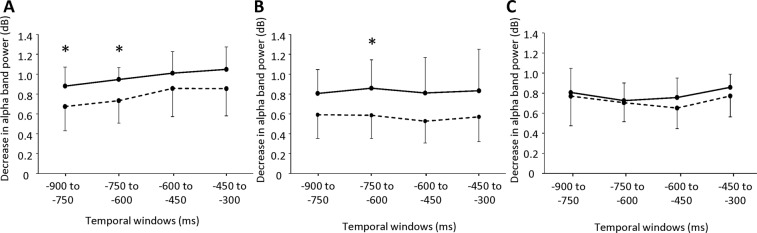
The decrease in alpha-band power observed in the left and right Brodmann’s area (BA) 6 in Tasks (**A**–**C**). (**A**) Decrease in alpha-band power observed in the left (solid line) and right (dotted line) BA 6 in Task A. (**B**) Decrease in alpha-band power observed in the left (solid line) and right (dotted line) BA 6 in Task B. (**C**) Decrease in alpha-band power observed in the left (solid line) and right (dotted line) BA 6 in Task C. The alpha-band power from −900 to −750 ms, −750 to −600 ms, −600 to −450 ms, and −450 to −300 ms before the button-press were calculated relative to that in the baseline period, which corresponded to the last 300 ms of presentation of the fixation. *P < 0.01, paired *t*-test with Bonferroni correction between the left and right BA 6 at each temporal window in Tasks (**A**–**C**).

Two-way repeated-measures ANOVA was performed to assess changes in the decrease of alpha-band power prior to button-press between left and right BA 6 in Tasks A, B, and C (Fig. 3). Main effects of laterality were seen in Task A [F(1, 8) = 15.027, P = 0.005] and Task B [F(1, 8) = 9.031, P = 0.017] (Fig. 3A,B). No main effect of laterality was evident in Task C [F(1, 8) = 2.211, P = 0.175] (Fig. 3C). The sample size necessary to detect the difference of the decrease of alpha-band power between the left and right BA 6 in Task C was calculated by using the software (i.e., G*Power). The alpha and beta error were set at 0.05 and 0.1, respectively, and the effect size was calculated based on the results of the two-way repeated-measure ANOVA performed for the difference of alpha-band power between the left and right BA 6 in Task A. The total sample size necessary to detect the difference of the decrease of alpha-band power between the left and right BA 6 in Task C was 4.

In Task A, the decrease of alpha-band power in the left BA 6 was greater than that in the right BA 6 in the temporal windows of −900 to −750 ms (*t*_8_ = 2.676; *P* = 0.028, paired t test with Bonferroni correction) and −750 to −600 ms (*t*_8_ = 3.690; *P* = 0.00613, paired t test with Bonferroni correction; Fig. 3A). In Task B, the decrease of alpha-band power in the left BA 6 was greater than that in the right BA 6 for the temporal window of −750 to −600 ms (*t*_*8*_ = 2.540; *P* = 0.0347, paired t test with Bonferroni correction; Fig. 3B).

## Discussion

The present study examined alterations in oscillatory neural activity as assessed by alpha-band power in the time period before perception of the intention to press the button when a volitional button-press was required (Task A) and those before the perception of fatigue when button-press was required when the fatigue sensation was perceived (Task C). The decrease of alpha-band power in the left BA 6, which includes the SMA, observed before perception of the intention to press the button in Task A was greater than those before perception of fatigue in Task C. In addition, although the decreases in alpha-band power in the left BA 6 were greater than those in the right BA 6 in the temporal windows of −900 to −750 ms and −750 to −600 ms prior to the onset of button-press in Task A, no difference was evident between decreases in alpha-band power in the left BA 6 and the right BA 6 in Task C.

The intention to move is reportedly perceived around 200 to 300 ms before the onset of movement^13–15^. In the present study, only those trials in which the timing of perception of the intention to move (Task A), timing of recognition of the answer (Task B), and timing of the perception of fatigue (Task C) were within the temporal window of −300 to 0 ms from the onset of the button-press were included in analyses. Since the numbers of trials excluded from the analyses did not differ between Tasks A and B or between Tasks A and C (Table 1), the delay between perception and button-press did not seem to differ among tasks.

Since there have been reports that the left SMA is involved in arithmetic processing^18,19^ and that the decrease in alpha-band power is related to the processing of information in that brain region^24,32^, the decrease in alpha-band power was expected to be observed in the left BA 6 prior to determination of the answer. In fact, the decrease to alpha-band power in the left BA 6 was greater than that in the right BA 6, confirming that a difference in the level of decrease in alpha-band power between left and right BA 6 was detectable with our analytical methods using data from MEG. Of course, the possibility remains that part of this decreased alpha-band power in the left BA 6 observed in Task B involved neural activity related to the button press, as in Task A. However, in this study, clarifying the cause of the decreased alpha-band power observed in Task B was difficult.

The decrease of alpha-band power in the left BA 6 before perception of the intention to move was greater than that in the right BA 6 in Task A. This is consistent with previous MEG and electrophysiological studies showing that the neural activity preceding voluntary hand movements were observed in the SMA contralateral to the hand moved^16,17,33^. We presumed that this decrease in alpha-band power observed from −900 to −300 ms before the onset of the button-press represented neural activity preceding perception of the intention to move in Task A. The question in the present study was therefore whether the decrease in alpha-band power prior to perception of fatigue in the left BA 6 was greater than that in the right BA 6 in Task C. The result was that the level of decrease in alpha-band power in the left BA 6 was not different from that in the right BA 6 in Task C. In addition, the level of the decrease in alpha-band power in the left BA 6 in Task C was lower than that in Task A. These observations showed that the decrease in alpha-band power prior to perception of fatigue was absent in Task C.

The RP and the decrease in alpha-band power have reportedly been observed in the time period preceding perception of the intention to move when voluntary movement is required^13–17^. Numerous studies into the RP preceding the movements have been conducted, and the RP has been proposed to be related to preparation for the upcoming movement, making the decision to move, anticipation of the movement, and so on^11,12,34,35^. The RP has also been proposed to play a role in affecting the propensity to act when voluntary movement is required and reflects neither preparation for movement nor the decision process to initiate the movement^12^. In contrast, a limited number of studies have regarded the alterations in oscillatory neural activity preceding the onset of movements, and the relevance of alterations in oscillatory neural activity to the RP is not well understood. Although the origin of the decrease in alpha-band power observed prior to the onset of movements seems to differ from that of the RP because the temporal and spatial distributions of the decreased alpha-band power differ from those of the RP^34,36^, the decreases in alpha- and beta-band power preceding the movement have been proposed to be related to the release of the idling state of the brain region in preparation for activation^15^.

Although interpretation of the role of the decreased alpha-band power observed before the onset of movement remains contentious, as described above, taking the fact that the contralateral SMA has been related to the preparation for and/or initiation of movement^37,38^, our findings that the decrease to alpha-band power in the left BA 6 was not observed before perception of fatigue in Task C seems to suggest that the button press was not prepared, decided, or anticipated before perception of fatigue, or that no preceding neural activity affecting the propensity to press the button was needed in Task C, supporting the possibility that perception of fatigue represents the direct cause for initiating the button-press. In line with this interpretation, the prolonged negativity assessed by EEG preceding the onset of movement was reportedly not observed when the movement was triggered by a random cue of unpredictable timing^39^.

Some limitations to the present study must be considered. First, we focused on those trials in which the timing of the perception of the intention to move or the perception of fatigue was within the temporal window of −300 to 0 ms from the onset of the button-press, based on the previous studies^13–15^. To ensure exclusion of those trials in which perception occurred more than 300 ms before the button-press, the letter presented on the center of the screen to assess timing of the perception was updated every 450 ms, so that recognition of the letter presented at the timing of the perception was easy enough for our participants. Thus, although it was possible to select trials in which the timing of perception was within the temporal window of −300 to 0 ms from the onset of the button-press, identification of the precise timing (to the nearest millisecond) at which this perception occurred in our present study was not possible. Second, the button press was performed using the right hand. Since the temporal and spatial distributions of decreases in oscillatory brain activity induced by self-paced hand movement has been reported to differ between right- and left-hand movements in right-handed participants^40^, adding a condition with a left-hand button press may increase the understanding of alterations in oscillatory brain activity preceding movements. Third, although our experiments were performed on 19 participants, the number of participants whose data were analyzed was 9. However, since we confirmed that the total sample size necessary for the two-way repeated-measure ANOVA to detect the difference of the decrease of alpha-band power between the left and right BA 6 in Task C was smaller than 9, our result that the decrease of alpha band power in the left BA 6 was not observed compared with that in the right BA 6 in Task C is not due to the lack of statistical power. Fourth, only the alteration of alpha band power in the left and right BA 6 was assessed in our present study. It is of great interest whether the alterations in neural activity in the brain regions other than the BA 6 was observed. However, since our present study was designed only to assess the alteration of alpha band power observed in the right and left BA 6, the number of participants whose data were analyzed was too small to conduct analyses assessing the alterations in neural activity in several other brain regions using such as statistical parametric mapping. Future studies are needed on this point.

In conclusion, in the present study, a decrease in alpha-band power in the left BA 6 was not observed before perception of fatigue sensation in Task C, but was observed before perception of the intention to move in Task A. These findings seem to suggest that perception of fatigue sensation represents the direct cause of the decision to initiate the button-press. Although further studies are needed to conclude whether the decision considered to have been made based on the perception of fatigue has actually already been made before conscious perception of fatigue, this study should motivate investigation of the neural mechanisms related to subjective feelings and help understand the functional roles of the neural activity preceding the movement.

## Data Availability

All data is available in the manuscript. Code for experimental task can be received by e-mail upon request.
